# KIR2DL5: An Orphan Inhibitory Receptor Displaying Complex Patterns of Polymorphism and Expression

**DOI:** 10.3389/fimmu.2012.00289

**Published:** 2012-09-17

**Authors:** Elisa Cisneros, Manuela Moraru, Natalia Gómez-Lozano, Miguel López-Botet, Carlos Vilches

**Affiliations:** ^1^Immunogenetics-HLA, Immunology Department, Hospital Universitario Puerta de HierroMajadahonda, Spain; ^2^Hospital del Mar Research Institute (IMIM), Universitat Pompeu FabraBarcelona, Spain; ^3^Immunology Unit, Universitat Pompeu FabraBarcelona, Spain

**Keywords:** gene polymorphism, human NK cells, inhibitory receptors, KIR, NK cell receptors, transcription

## Abstract

A recently developed anti-KIR2DL5 (CD158f) antibody has demonstrated KIR2DL5 expression on the surface of NK and T lymphocytes, making it the last functional KIR identified in the human genome. KIR2DL5 belongs to an ancestral lineage of KIR with Ig-like domains of the D0-D2 type, of which KIR2DL4, an HLA-G receptor, is the only other human member. Despite KIR2DL4 and KIR2DL5 being encoded by genes with similar domain usage, several KIR2DL5 functions resemble more closely those of KIR recognizing classical HLA class I molecules – surface-expressed KIR2DL5 inhibits NK cells through the SHP-2 phosphatase and displays a clonal distribution on NK and T lymphocytes. No activating homolog of KIR2DL5 has been described in any species. The genetics of KIR2DL5 is complicated by duplication of its gene in an ancestor of modern humans living ∼1.7 million years ago. Both *KIR2DL5* paralogs have undergone allelic diversification; the centromeric gene is most often represented by alleles whose expression is silenced epigenetically through DNA methylation, thus providing a natural system to investigate the regulation of *KIR* transcription. The role of KIR2DL5 in immunity is not completely understood, in spite of different attempts to define its ligand. Here we revisit the most relevant characteristics of KIR2DL5, an NK-cell receptor possessing a unique combination of genetic, structural, and functional features.

## Introduction

KIR2DL5 (CD158f) is the most recently described human KIR expressed on NK and T lymphocytes (Estefanía et al., 2007), for which no ligands have yet been identified. It belongs to an ancestral lineage of KIR with Ig-like domains of the D0-D2 type, whose only other member is KIR2DL4, an HLA-G receptor (Rajagopalan, 2010; Rajagopalan and Long, 2012). Although the *KIR2DL5* and *KIR2DL4* genes encode proteins with a similar domain organization, distinct structural features make several KIR2DL5 functions resemble more closely those of KIR recognizing classical HLA class I molecules (Table 1).

**Table 1:** **Structural, genetic, and functional features of KIR2DL5 in comparison with other human KIR**.

	KIR2DL5	KIR2DL4	KIR2DL1	KIR3DL1
Ig-like domains	D0-D2	D0-D2	D1-D2	D0-D1-D2
No. of exons encoding Ig-like domains	2	2	2+ pseudoexon	3
Charged residue in transmembrane	No	Yes	No	No
Tyrosine-based signaling motifs	1 ITIM, 1 ITSM	1 ITIM	2 ITIM	2 ITIM
Signaling molecules	SHP-2 > 1	Fc_ε_Rγ, DNA-PKcs*	SHP-1 > 2	SHP-1 > 2
Function	Inhibition	IFNγ secretion, inhibition?	Inhibition	Inhibition
Transcription in NK cells	Clonal	Ubiquitous	Clonal	Clonal
Ligand	Unknown	HLA-G	HLA-C	HLA-A/B
Copy number variation	++	±	+	±**
Conservation in primates	++	+++	−	±

*(Kikuchi-Maki et al., 2005; Rajagopalan, 2010).

**Most haplotypes lacking KIR3DL1 have its KIR3DS1 allotype.

The 9.3-kbp *KIR2DL5* gene was identified in 2000 by amplification of genomic DNA with oligonucleotide primers recognizing conserved *KIR* regions (Vilches et al., 2000b) and analysis of the first sequenced *KIR* haplotype (Wilson et al., 2000). Exon-walking and RACE strategies isolated the complete *KIR2DL5* coding region, an open reading frame of 1128 bp encompassing eight exons organized similarly to those of *KIR2DL4* – they both lack the fourth exon coding for the D1 Ig-like domain in all other KIR, and encode cytoplasmic tails 20–39 amino acids longer than other human inhibitory KIR (Vilches and Parham, 2002).

This structure is conserved in *KIR2DL5* orthologs identified in common and pigmy chimpanzees, gorillas, and orangutans (Khakoo et al., 2000; Rajalingam et al., 2004; Guethlein et al., 2012). Genomic and complementary DNA clones isolated from other Old World primates resemble human *KIR2DL5* in part of its sequence or in the domain organization, but true functional orthologs appear to be restricted to hominoids (Hershberger et al., 2001, 2005; Sambrook et al., 2005; Bimber et al., 2008; Abi-Rached et al., 2010; Palacios et al., 2011). No activating homolog of KIR2DL5 has been described in any species, human KIR2DS5 being homologous to HLA-C-specific KIR.

## Genetic Organization: Two *KIR2DL5* Genes Subjected to Extensive Copy Number Variation

KIR2DL5 is highly polymorphic, like other KIR, and it epitomizes the copy number variation that is a hallmark of the KIR complex. Non-mendelian inheritance and different relative locations of the two most common variants seen in Caucasoids demonstrated that *KIR2DL5* alleles belong to two series encoded by different loci (Vilches et al., 2000a; Gómez-Lozano et al., 2002). These loci, designated officially *KIR2DL5A* and *KIR2DL5B*, are now often referred to with the suffixes T and C, for their location in the telomeric and the centromeric intervals of the KIR complex, respectively (Marsh et al., 2003; Pyo et al., 2010; Parham et al., 2012).

### Linkage to *KIR2DS3S5* in *KIR*-B haplotypes

**Figure 1:**
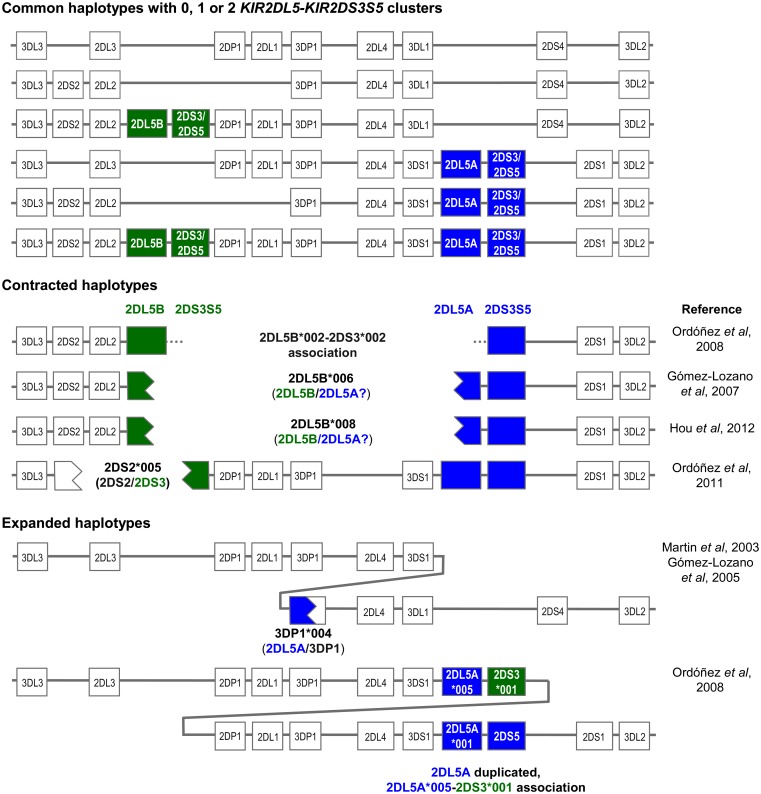
**Copy number variation and allelic polymorphism of *KIR2DL5*-*KIR2DS3S5* clusters contribute substantial diversity to *KIR* haplotypes**.

Both the centromeric and the telomeric *KIR2DL5* loci are followed by the paralogs of a duplicated *KIR2DS3S5* gene, each of which encodes different alleles of the activating KIR 2DS3 and 2DS5, now considered allotypes of each other (Ordóñez et al., 2008; Hou et al., 2010; Pyo et al., 2010). Thus, the centromeric and the telomeric parts of many *KIR*-B haplotypes are marked by different *KIR2DL5*-*KIR2DS3S5* clusters (Figure 1). The common centromeric sequence KIR2DL5B*002 is associated with KIR2DS3*001, whereas other *KIR2DL5B* alleles (see below) tend to associate in Black populations with several *KIR2DS5* alleles (Hou et al., 2010). On the telomeric side, the predominant *KIR2DL5A* alleles, *001, and *005, are linked with KIR2DS5*002 and KIR2DS3*002, respectively. At its 5’ end, *KIR2DL5B* is normally flanked by *KIR2DL2*, whereas *KIR2DL5A* is preceded by *KIR3DS1* (Vilches et al., 2000a; Pyo et al., 2010).

### Duplication of *KIR2DL5* is specific to humans

The *KIR2DL5* duplication has not been seen in other primates, and is possibly specific to humans. Pyo et al. (2010) estimated that an ancestral *KIR2DL5*-*KIR2DS3S5* group duplicated ca. 1.7 million years ago, and proposed several models for subsequent diversification through point mutation and recombination. The duplication, seen in all races, is now fixed in our species. However, not every human carries two (or one) *KIR2DL5*-*KIR2DS3S5* clusters, because each is subjected to presence/absence variation, with all A haplotypes and one centromeric B haplotype lacking these genes (Figure 1).

### Expanded and contracted *KIR* haplotypes generated by recombination in the *KIR2DL5-KIR2DS3S5* cluster

On the other hand, presence of two highly homologous sequence segments in two different parts of the KIR complex has facilitated subsequent asymmetric (i.e., non-allelic) homologous recombination resulting in contracted and expanded haplotypes (one of them with a third *KIR2DL5* locus), often carrying fusion genes or alleles, as represented in Figure 1 (Gómez-Lozano et al., 2003, 2005, 2007; Martin et al., 2003; Ordóñez et al., 2008, 2011; Hou et al., 2012). In contracted haplotypes lacking the central framework KIR genes, assignment of *KIR2DL5* and *KIR2DS3S5* to the centromeric or the telomeric sides is somewhat arbitrary.

## *KIR2DL5* Allelic Polymorphism

### The *KIR2DL5* coding region

*KIR2DL5* is represented in the Immuno Polymorphism Database (v2.4.0) by 15 *KIR2DL5A* and 25 *KIR2DL5B* alleles (Robinson et al., 2010). Nineteen polymorphic sites have been found within the 1125-bp coding region, of which 11 are non-synonymous. Twelve nucleotide substitutions occurring in exons 3 and 5 create seven amino acid replacements in the extracellular Ig-like domains (Table 2), which may reflect balancing selection having favored polymorphisms that could modulate avidity or specificity in the interaction of KIR2DL5 with unknown ligands. Du et al. (2008) pointed out, however, that many polymorphisms fall out of predicted ligand-interacting loops of the Ig-like domains. Of note, a single polymorphism in exon 1 distinguishes all *KIR2DL5A* from all *KIR2DL5B* alleles, whilst many substitutions are shared by alleles of both loci (Table 2). An extensive exchange of genetic material between the centromeric and the telomeric *KIR2DL5* loci has taken place during human evolution, as eloquently illustrated by two allele pairs (one from each locus) and a four-allele group (two from each locus) encoding identical mature polypeptides and differing only in their signal peptides. Among 65 additional polymorphisms occurring in *KIR2DL5* introns (not shown), none alters its splicing sites.

**Table 2:** **Comparison of the deduced primary structures and surface expression profiles of *KIR2DL5* alleles**.

*2DL5 allele*	Leader peptide exons 1–2		D0 domain exon 3			D2 domain exon 5				Stem exon 7	Cyt. exon 9	Surface expression detectable with UP-R1*
	−16 Ile	−1 Thr	16 Ala	78 His	95 Val	114 Arg	152 Asn	167 Gly	174 Gly	215 Arg	284 Val	
A*001	–	–	–	–	–	–	–	–	–	–	–	Yes
B*008	Val	–	–	–	–	–	–	–	–	–	–	na
A*012	–	Pro	–	–	–	–	–	–	–	–	–	(Yes)
B*006	Val	Pro	–	–	–	–	–	–	–	–	–	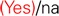
A*015	–	–	–	–	–	Cys	–	–	–	–	–	?
B*013	Val	–	–	–	–	Cys	–	–	–	–	–	na
A*005	–	–	–	–	–	–	Asp	–	Ser	–	–	No
B*002	Val	–	–	–	–	–	Asp	–	Ser	–	–	na
A*014	–	–	–	–	–	–	–	Arg	–	–	–	?
B*004	Val	–	Thr	–	–	–	–	–	–	–	–	na
B*007	Val	–	–	–	Met	–	–	–	–	–	–	na
B*003	Val	Pro	–	–	Met	–	–	–	–	–	–	?
B*011	Val	Pro	–	–	–	–	–	–	–	–	Ile	na
B*009	Val	–	–	–	–	–	Asp	–	Ser	Leu	–	na
B*010	Val	Pro	–	–	–	–	Asp	–	Ser	–	–	na
B*016	Val	–	–	Tyr	–	–	Asp	–	Ser	–	–	na

**Yes: surface expression demonstrated by flow cytometry with UP-R1; No: lack of staining with UP-R1 demonstrated in KIR2DL5A*005 cells; (Yes): surface expression and staining with UP-R1 is predictable, according to the allele primary structure and promoter type, but has not been assessed; ?: surface expression and staining with UP-R1 can not be predicted in a transcribed allele, due to polymorphisms in the ectodomain; na: not applicable due to demonstrated or likely lack of transcription; (Yes)/na: different variants of allele KIR2DL5B*006 have functional or non-functional promoters (Table 3). *KIR2DL5A* alleles are shown in 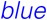, and *KIR2DL5B* ones, in 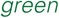. Allele order has been arranged to highlight patterns of homology between alleles*.

### Polymorphism in the *KIR2DL5* proximal promoter region

The regulatory regions upstream of the *KIR2DL5* genes are even more polymorphic – the three first known *KIR2DL5* alleles are distinguished by 20–32 nucleotide substitutions in the 1.2-Kbp region immediately 5′ of their start codon (1.6–2.5% variation). A neighbor-joining phylogenetic tree based on the nucleotide sequences of this region sorts *KIR2DL5* alleles into three well-differentiated lineages. One of them includes all and only *KIR2DL5A* alleles; a second lineage comprises multiple *KIR2DL5B* alleles, of which 2DL5B*0020101 is the prototype; and the third cluster is formed by KIR2DL5B*003 and *00602 (Du et al., 2008). We will refer herein to these clusters as promoters of types I, II, and III. The origin of this divergence, of profound functional importance (alleles controlled by type II promoter are not transcribed), has not been explained.

### Distribution of *KIR2DL5* alleles

*KIR2DL5* is present in all human populations at frequencies ranging from 26 to 86%, but the distributions of the two paralogs and their allotypes are uneven. Whereas *KIR2DL5A* and *KIR2DL5B* predominate in Mongoloid and Black populations, respectively, they have similar frequencies in Caucasoids. Alleles KIR2DL5A*001, B*002, and A*005 are widely distributed, accompanied by B*006 in Blacks, who retain the highest *KIR2DL5* diversity, and constitute the only human group in which *KIR2DL5* alleles controlled by the third type of promoter are not rare (Vilches et al., 2000a; Gómez-Lozano et al., 2007; Du et al., 2008; Middleton et al., 2008; Mulrooney et al., 2008; Hou et al., 2010; González-Galarza et al., 2011 and our own unpublished results).

### *KIR2DL5* and disease

Data on possible implication of *KIR2DL5* copy number variation and polymorphism in susceptibility to disease are scarce. Complex polymorphism and strong linkage disequilibrium with neighboring *KIR* genes complicates evaluating the individual role of *KIR2DL5* as a risk or a protective factor. Search of the PubMed database with the term “KIR2DL5” in June 2012 retrieved 16 citations describing significant deviations of the gene frequency in different diseases and clinical situations (not shown). Among them, only an association between ankylosing spondylitis and presence of *KIR2DL5* in the genome of Asian patients has been replicated (Díaz-Peña et al., 2008; Jiao et al., 2008, 2010).

### *KIR2DL5* genotyping

*KIR2DL5* polymorphism has been explored using PCR with sequence-specific primers (SSP) or oligonucleotide-probe hybridization (2008), methods that reliably identify common alleles (Gómez-Lozano and Vilches, 2002; Gómez-Lozano et al., 2007; González et al., 2008). Sequence-based typing (SBT) and mass spectrometry methods that enable studying the entire *KIR2DL5* sequence have led to identification of multiple new alleles (Houtchens et al., 2007; Du et al., 2008; Mulrooney et al., 2008; Hou et al., 2010). However, existence of two *KIR2DL5* loci poses extra difficulties to genotyping: firstly, because a person having the two loci on both chromosomes may have up to four different *KIR2DL5* sequences; secondly, because the alleles of each locus share many single-nucleotide polymorphisms (SNPs). Knowing the phase of *KIR2DL5* SNPs is essential for locus/allele assignment, but this is hindered by the hundreds or thousands of base-pairs separating many individual polymorphism (e.g., the only locus-specific SNP in exon 1 is ca. 3 Kbp apart from those in exon 5). The published methods can make tentative assignments of reasonable reliability on samples derived from populations in which the *KIR2DL5* allele distribution has been previously investigated in depth, but none of them can assign unambiguously all possible *KIR2DL5* genotypes. Separation of *KIR2DL5* alleles by locus-specific long-range PCR, followed by probe hybridization or enzymatic sequencing, and long reads of individual DNA molecules by second-generation sequencing are promising strategies for accurate KIR2DL5 genotyping, which remains currently a challenge.

## *KIR2DL5* Expression

### Gene transcription

The fact that highly similar *KIR2DL5* coding sequences are controlled by three structurally divergent forms of promoter has profound functional consequences, constituting a valuable natural experiment that provides major insight into the complex regulation of *KIR* transcription. Of the *KIR2DL5* alleles whose transcription has been investigated, those controlled by type I or type III promoters feature variegated patterns of expression; whilst mRNA of alleles controlled by type II promoters is undetectable (Vilches et al., 2000a; Gómez-Lozano et al., 2007). No single exception to this rule has ever been described; furthermore, the *KIR3DP1* pseudogene, also controlled by a type II promoter, is transcriptionally silent too, with a key exception: as the empirical rule predicted, KIR3DP1*004, which gained a type I promoter through recombination with *KIR2DL5A*, is an expressed allele (Vilches et al., 2000b; Gómez-Lozano et al., 2005).

Consistent with the epigenetic regulation of *KIR* genes (Santourlidis et al., 2002; Chan et al., 2003), lack of transcription of the silent KIR2DL5B*002 allele correlates with a hypermethylated status of CpG islands in its promoter. Furthermore, pharmacological DNA demethylation of cultured NK cells suffices for restoring KIR2DL5B*002 transcription, demonstrating that only an epigenetic mechanism prevents its expression (Gómez-Lozano et al., 2007). In agreement with this are studies of transiently transfected promoters controlling a reporter gene, an *in vitro* situation in which epigenetic regulation is not relevant. In this setting, the promoters of naturally silent *KIR2DL5* alleles tend to show similar or higher activities than functional *KIR* alleles (Gómez-Lozano et al., 2007; Mulrooney et al., 2008).

Among the sequence patterns that distinguish the three types of *KIR2DL5* promoter, only two linked SNPs at nucleotides 97 and 84 upstream of the start codon correlate completely with the expression pattern: GA is seen in transcribed alleles, and AG in silent ones (Table 3). Nucleotide −97G lies within a TGTGGT motif that provides a core binding site for the RUNX family of transcription factors (Vilches et al., 2000a). RUNX3 is recruited from nuclear extracts of NK cells by probes derived from *KIR2DL5* alleles having an intact motif, but not by those carrying the −97G > A mutation (Gómez-Lozano et al., 2007). In support of an essential role for RUNX in KIR expression is conservation of its binding motif in all human *KIR* with clonal transcription (Trompeter et al., 2005; van Bergen et al., 2005; Presnell et al., 2006); and demonstration that two redundant RUNX binding sites, highly conserved in primates, are possibly essential for expression of *KIR2DL4*, a gene that is transcribed ubiquitously in NK cells (Presnell et al., 2012).

**Table 3:** **Comparison of the proximal promoter sequences and transcription profiles of *KIR2DL5* alleles**.

*KIR2DL5* allele	Promoter region						Promoter type	Transcription
	−104 G	−97 G	−84 A	−27 C	−23 C	−10 C	
A*0010101–00105	–	–	–	–	–	–	I	+
A*0050101/03–04	–	–	–	–	–	–	I	+
A*01201/02	–	–	–	–	–	–	I	(+)
A*0050102	A	–	–	–	–	–	I	(+)
B*0020101–03/05/07, *0070101	A	A	G	–	T	–	II	−
B*004, *0080101/00802, *009, *01301/02	A	A	G	–	T	–	II	−
B*00601/03	A	A	G	–	–	–	II	−
B*0020104, *00202, *0080102, *010, *011	A	A	G	–	–	–	II	(−)
B*003	A	–	–	T	–	T	III	+
B*00602	A	–	–	T	–	T	III	(+)

Transcription of the alleles shown in bold face has been assessed experimentally; in parentheses are shown predictions for groups of alleles of which no member has been studied by RT-PCR. The promoter regions of *KIR2DL5* alleles A*014, A*015, B*0020106, B*0070102, B*01303, and B*016 have not been sequenced.

*KIR* transcription is controlled not only by a proximal promoter, but also by the complex interaction of additional regulatory elements (Cichocki et al., 2011). In brief, a distal, non-tissue-specific promoter element located ∼1.1 Kbp upstream of the KIR start codon has been suggested to induce histone modifications that facilitate subsequent function of the proximal promoter. The latter is actually bidirectional – reverse transcripts derived from it have been proposed to repress KIR expression and favor epigenetic silencing, whilst predominance of forward transcription would result in KIR expression. Finally, an additional reverse promoter element in intron 2 appears to function in early NK-cell progenitors. It has been suggested that the RUNX role might be to down-regulate the antisense promoter activity during NK-cell ontogeny, thus favoring a local open chromatin conformation at the bound *KIR* gene, and its subsequent expression in the mature cell (Davies et al., 2007; Cichocki et al., 2011). Consistent with this hypothesis is that the only reverse *KIR* transcripts detected in CD56^bright^ NK cells (possible precursors of KIR^+^CD56^dim^ cells) are those derived from genes with promoters lacking the RUNX binding site – KIR2DL5B*002 and *KIR3DP1* (Davies et al., 2007).

Other locus- and allele-specific polymorphisms of these regulatory elements influence KIR transcription and may also help us understand mechanisms controlling KIR2DL5 expression. For instance, a Ying Yang-1 (YY1) binding site conserved in many proximal KIR promoters is mutated in *KIR* *2DL1*, *2DS1/S3/S5*, and all *KIR2DL5* alleles, which correlates with enhanced reverse transcription (Davies et al., 2007; Li et al., 2008). This phenomenon may induce low forward activity of the *KIR2DL5* promoter, which might be associated with the receptor being generally expressed at low levels on the surface of only small proportions of NK cells (Estefanía et al., 2007). Likewise, disruption of the Sp1 site in the promoter of the expressed allele KIR2DL5B*003 (−27C > T, Table 3) decreases its forward activity *in vitro* and has been proposed to reduce its expression on NK cells (Li et al., 2008), which needs experimental confirmation.

Of possible interest, none of the transcriptionally silent *KIR2DL5B* alleles bear structural abnormalities in their reading frames (in contrast with other human *KIR*, no null *KIR2DL5* alleles have yet been identified; Vilches et al., 2000a; Robinson et al., 2010). The fact that *KIR2DL5B* generally retains an intact structure could mean that inactivation of its expression is evolutionarily recent (the mutated RUNX site is not seen in other hominoids, personal communication of Libby Guethlein, Stanford University); or that the gene still serves an unknown function.

### Cell surface expression

Generation of a specific monoclonal antibody (clone UP-R1) enabled us to characterize KIR2DL5 surface expression (Estefanía et al., 2007). Like most other KIR (and contrasting with the non-clonal expression of KIR2DL4), KIR2DL5 features a variegated pattern on the surface of CD56^dim^ NK cells and on T lymphocytes from peripheral blood, in agreement with the clonal distribution seen by reverse transcription (RT) PCR in mRNAs isolated from NK- and T-cell clones (Vilches et al., 2000b; Estefanía et al., 2007). The proportion of NK cells expressing KIR2DL5 tends to be lower than 10% in most healthy individuals. That proportion is even lower in T lymphocytes, of the CD8 subset in their vast majority. The receptor density on the surface, as assessed by the median fluorescence intensity (MFI) value in flow cytometry with mAb UP-R1, is also lower than for several other KIR in resting lymphocytes, but it increases, to a lesser extent, upon expansion in presence of IL-2 and lymphoblastoid cell lines (our own observation). These features might owe to weak promoters controlling the transcription of functional *KIR2DL5* alleles.

Interestingly, higher numbers of KIR2DL5^+^ cells (20% of total NK lymphocytes) have been reported in a TAP-deficient woman. Furthermore, the phenotype of this patient also differed from that of most TAP-deficient individuals in her resting NK cells retaining cytotoxic capacity against allogeneic targets without pre-activation (Zimmer et al., 2009). The exact mechanisms determining this behavior remain to be ascertained.

Analysis of bulk and cloned NK cells by flow cytometry and RT-PCR reveals no coordinated expression of KIR2DL5 with other KIR, but rather an apparently random distribution (Vilches et al., 2000b; Estefanía et al., 2007). Importantly, a minority of NK cells expresses KIR2DL5 but neither other inhibitory KIR, nor the inhibitory lectin-like receptor NKG2A. Existence of this subpopulation is consistent with a capacity of KIR2DL5 to license NK cells, but this has not been demonstrated functionally. Also lacking are studies on possible patterns of co-expression of KIR2DL5 and LILRB1, the third lineage of inhibitory MHC class I receptors expressed by human NK cells.

Allelic polymorphism is essential for understanding the different patterns of KIR2DL5 expression (Table 2). Transcriptionally silent alleles are, obviously, undetectable on the cell surface by definition. Furthermore, only allele KIR2DL5A*001 has been formally demonstrated to be expressed on the cell surface. In contrast, NK cells transcribing allele KIR2DL5A*005 are not stained by mAb UP-R1 in flow cytometry (Gómez-Lozano et al., 2007). Whether this is due to a lack of surface expression, to the UP-R1 epitope being altered in KIR2DL5A*005 by its D2-domain polymorphisms, or to a combination of both factors, has not yet been elucidated. Surface expression and recognition by UP-R1 of other transcribed *KIR2DL5* alleles has, to the best of our knowledge, never been evaluated. Amongst other transcriptionally active *KIR2DL5* alleles, A*012 and B*00602 code for mature polypeptides identical to A*001, therefore they are predictably surface-expressed and detected by UP-R1; whereas expression and UP-R1 recognition of KIR2DL5B*003, A*014, and A*15 (each bearing one amino acid replacement in the Ig-like domains in comparison with A*001) needs to be tested empirically (Table 2).

## KIR2DL5 Function

### KIR2DL5 inhibits NK cells

KIR2DL5 is predominantly expressed on the cell surface as a glycosylated monomer of ∼60 kDa (Estefanía et al., 2007). Its cytoplasmic tail contains one canonical (VxYxxL) immunoreceptor tyrosine-based inhibitory motif (ITIM) separated by 24 amino acid residues from an atypical ITIM sequence (TxYxxL) similar to the immunoreceptor tyrosine-based switch motifs (ITSM) seen in 2B4, SLAM, and other receptors (Vilches et al., 2000b; Yusa et al., 2004). The latter motif, not seen in other human KIR, does not confer upon KIR2DL5 the capacity to recruit and signal through the SLAM-associated protein (SAP), but it is conserved in KIR2DL5 orthologs of other hominoids (Rajalingam et al., 2004; Yusa et al., 2004).

Since the KIR2DL5 ligand is unknown, its actual inhibitory character in physiological conditions has not been explored. Cross-linkage of naturally expressed KIR2DL5 inhibits NK-cell cytotoxicity against mAb-coated P815 target cells to an extent comparable to that seen with the “classical” KIR 3DL1 (Estefanía et al., 2007). This result is in agreement with that obtained previously using NK92 cells transduced with a chimera containing a KIR3DL1 ectodomain fused to the KIR2DL5 cytoplasmic tail; such chimera, however, displayed a lower capacity to inhibit NK92-target conjugation than full-length KIR3DL1 (Yusa et al., 2004). Based on the results obtained with a mutated KIR3DL1/2DL5 chimera, Yusa et al. (2004) proposed that the canonical KIR2DL5 ITIM, but not its ITSM-like motif, is essential for its inhibitory capacity in transduced NK92 cells.

Experiments performed independently on transduced NK92 cells and on NK cells expressing endogenous KIR2DL5 demonstrated that the phosphorylated receptor recruits the Src homology region 2-containing protein tyrosine phosphatase 2 (SHP-2) preferentially over SHP-1 in comparison with other KIR (Yusa et al., 2004; Estefanía et al., 2007). Furthermore, the inhibitory effect of the KIR2DL5 tail in transduced NK92 cells was prevented by a dominant-negative (DN) SHP-2, but only to a lesser extent by DN SHP-1 (Yusa et al., 2004). The importance of KIR2DL5 using predominantly a SHP-2-dependent pathway for its function has not been explored in depth.

### KIR2DL5, an orphan receptor

Demonstration that KIR2DL5 is a surface-expressed glycoprotein capable of inhibiting cytotoxic lymphocytes suggested that this molecule participated in NK-cell mediated defense according to the missing-self model. Such a possibility was reinforced by identification of NK cells which express KIR2DL5 and lack all other detectable inhibitory KIR and NKG2A, and it implies existence of a cellular ligand, possibly expressed in physiological conditions. Enhanced KIR2DL5 expression and retention of NK-cell cytotoxicity in a TAP-deficient patient (Zimmer et al., 2009) suggest that she possibly expressed a ligand capable of licensing KIR2DL5^+^ NK cells.

As a first approach to investigate expression of a KIR2DL5 ligand, we made a fusion protein containing the KIR2DL5 ectodomains and the Fc of human IgG1. The fusion protein, along with positive and negative controls (KIR2DL1-, KIR2DL2-, and non-fused Fc constructs kindly donated by Dr. Eric Long), was produced in human embryonic kidney (HEK)-293T cells, and used in indirect flow cytometry experiments on multiple cell lines grown *in vitro*. In these experiments, we observed a dull staining of, essentially, every human cell line of hematopoietic origin. Such staining seemed independent of the cells HLA allotypes; furthermore, it was apparently not affected by lack of surface HLA expression in mutant cell lines (results not shown). However, the variably low signal-to-noise ratios with which the positive controls often stained cells expressing their known ligands, and the variable behavior of different batches of fusion proteins of known specificity indicated that the method did not attain sufficient sensitivity and consistency in our hands to allow screening for an unknown ligand in a series of heterogeneous cell types.

As an alternative approach of possibly higher sensitivity, we tried to apply the MHC-tetramer technology to build KIR2DL5 forms of higher avidity. The first codons of a *KIR2DL5* cDNA were adapted by site-directed mutagenesis to the codon usage bias of *Escherichia coli* (Nakamura et al., 2000), for higher protein yield; and the construct encoding the Ig-like and stem regions of KIR2DL5 was subcloned into the pGMT7 plasmid (a kind gift of Dr. Veronique Braud), which provided an in-frame recognition sequence for the BirA biotinylase at the carboxy-terminal end of the construct. Upon IPTG induction, the recombinant KIR2DL5 protein was efficaciously produced in strain BL21(DE3)pLysS. After purification from inclusion bodies, the KIR2DL5 ectodomain was solubilized in concentrated urea, refolded in an arginine/gluthation buffer, and biotinylated with BirA. However, the labeled KIR2DL5 protein could not be quantitatively recovered after molecular exclusion chromatography, apparently due to aggregation, even in presence of mild detergents like Chaps and Octyl-b-d-glycopyranoside.

The ability to identify KIR2DL5 with a novel specific monoclonal antibody opened new possibilities for studying the outcome of the interaction of KIR2DL5-positive NK lymphocytes with potential target cells. For instance, we attempted to study differential degranulation (assessed by CD107a expression) of KIR2DL5-positive and -negative NK cells against different target cells. However, several hindrances made this approach unpractical, including: low levels of degranulation induced in freshly isolated NK cells by many targets, which made it difficult to evaluate any further reduction attributable to inhibition through KIR2DL5; the low proportions of NK cells expressing KIR2DL5 in most donors, which do not readily increase during *in vitro* NK-cell expansion in response to lymphoblastoid cell lines. These studies indicated that use of cells homogeneously expressing KIR2DL5, and of a positive readout (rather than inhibition of another signal) are more promising approaches for screening the interaction of KIR2DL5 with potential ligand molecules. Knowing such interactions is essential for understanding the role of KIR2DL5 in immunity, and its importance for human health.

## Conflict of Interest Statement

The authors declare that the research was conducted in the absence of any commercial or financial relationships that could be construed as a potential conflict of interest.
